# Veratrum Viride, or American Hellebore, as an Arterial Sedative

**Published:** 1852-11

**Authors:** J. H. Woodcock


From the St. Louis Medical and Surgical Journal.

*Veratrum Viride or American Hellebore, as an Arterial Sedative.*—By J. H. Woodcock, M. D.

If not apprised already of it, I would wish to call your attention to an article lately brought to the notice of the profession here, as being very potent in controlling the circulation in disease of accelerated action of the heart and arteries.

My attention was called to the use of the article on yesterday, by the discoverer of it, Dr. Wesley C. Norwood, of Georgia, who in person handed me a small vial of the tincture for trial.

The article in question is a saturated Tr. of the veratrum viride, or green hellebore; made of the root of the plant. The herb grows abundantly in N. Carolina, Western New York, &c. It grows on hill sides in the vicinity of swamps. In appearance resembling the mullen, growing 4 or 5 feet high. I was unusually busy when the Dr. called, and failed to get either its botanical description or his address from him.

Supposing that your knowledge in this respect will enable you to find this plant in Missouri, is one of my leading reasons for addressing you on this subject, as the article is no doubt abundant with you.

Dr. Norwood read me some dozen letters from members of our profession, in S. Carolina and Georgia, affirmative of all he claims for the remedy. According to their united testimony, the Tr. given in 8 drop doses every three hours, will of a certainty reduce the pulsations from 130 per minute down to 95 or 80, in eight to twelve hours, thereby enabling us in puerperal fevers, pneumonia biliosa and other similar diseases, to break up the chain or diseased action like a charm. Dr. N. informed me that many cases descriptive of its successful use, were noticed in the Georgia Journal devoted to medicine.

The late edition of the United States Dispensatory has the following in reference to the medical properties and uses of the American hellebore. "In addition to its emetic action, which is often violent and long continued, it is said to increase most of the secretions, and when freely taken, to exercise a powerful influence over the nervous system, indicated by faintness, somnolency, vertigo, head-ache, dimness of vision, and dilated pupils. According to Dr. Osgood, it reduces the frequency and force of the pulse, sometimes when taken in full doses, as low as thirty-five strokes in the minute."

